# Autologous tumor lysate-loaded dendritic cell vaccination combined with Sunitinib for metastatic renal cell carcinoma

**DOI:** 10.1186/2051-1426-2-S3-P50

**Published:** 2014-11-06

**Authors:** Kazuhiro Kakimi, Hirokazu Matsushita, Yutaka Enomoto, Tohru Nakagawa, Haruki Kume, Yukio Homma

**Affiliations:** 1 The University of Tokyo Hospital, Tokyo, Japan; 2 Mitsui Memorial Hospital, Tokyo, Japan; 3 The University of Tokyo Hospital, Bunkyo-Ku, Japan

## Background

The antibodies that block immunological checkpoints have been successfully applied for the treatment of cancer. Similar effects are expected by molecular targeted therapy, which primarily aims to inhibit molecular pathways for tumor cell growth and survival. Such small molecular drugs may modulate the immune system, which raises the possibility that targeted therapy might be effectively combined with immunotherapy. Sunitinib, a tyrosine kinase inhibitor currently in use for the treatment of metastatic renal cell carcinoma (mRCC), has been reported to modulate immunosuppressive cells such as myeloid-derived suppressor cells (MDSCs) and regulatory T cells (Tregs). We conducted a clinical study of dendritic cell (DC)-based immunotherapy together with Sunitinib in mRCC patients in an effort to enhance immunotherapeutic efficacy by inhibiting immunosuppressive cells.

## Methods

Patients aged ≥20 years with advanced or recurrent mRCC who underwent nephrectomy were eligible for this study. Autologous tumor samples were obtained by surgery and used for preparing autologous tumor lysate. Leukapheresis was performed to obtain peripheral blood mononuclear cells (PBMCs). DCs were generated from adherent PBMCs in the presence of recombinant human granulocyte macrophage colony-stimulating factor (GM-CSF) (500 IU/ml) and IL-4 (500 IU/ml). Mature DCs were loaded with autologous tumor lysate by electroporation. Eight patients were enrolled in the study and received Sunitinib at a dose of 50 mg p.o. daily for 28 days followed by 14 days of rest. Tumor lysate-loaded DCs were administered subcutaneously every two weeks, with concomitant Sunitinib.

## Results

No severe adverse events related to vaccination were observed. Sunitinib decreased the frequencies of MDSCs in peripheral blood of 5 patients and of Tregs in 3. Tumor lysate-reactive CD4 or CD8 T cell responses were observed in 5 patients, 4 of whom showed decreased frequencies of Tregs and/or MDSCs. The remaining 3 patients who failed to develop tumor-reactive T cell responses had high levels of IL-8 in their sera and did not show consistent reductions in MDSCs and Tregs. The antigen spreading was observed; the production of antibodies against several cancer testis antigen was induced after DC vaccination. The median overall survival was 346 days and median progression-free survival was 164 days. One patient achieved a complete response, another patient had a partial response, 2 had stable disease and 3 had progressive disease according to the RECIST (one N/A).

## Conclusions

DC-based immunotherapy combined with Sunitinib is safe and feasible for patients with mRCC.

## Trial registration

Trial registration number: UMIN000002136

